# Pre-infection plasma cytokines and chemokines as predictors of HIV disease progression

**DOI:** 10.1038/s41598-022-06532-w

**Published:** 2022-02-14

**Authors:** Samukelisiwe Ngcobo, Refilwe P. Molatlhegi, Farzana Osman, Sinaye Ngcapu, Natasha Samsunder, Nigel J. Garrett, Salim S. Abdool Karim, Quarraisha Abdool Karim, Lyle R. McKinnon, Aida Sivro

**Affiliations:** 1 Centre for the AIDS Programme of Research in South Africa (CAPRISA), 719 Umbilo Road, Durban, South Africa; 2 Department of Medical Microbiology, University of KwaZulu-Natal, Durban, South Africa; 3 Department of Medical Microbiology, University of Manitoba, Winnipeg, Canada; 4 Department of Epidemiology, Columbia University, New York, NY USA; 5 Department of Medical Microbiology, University of Nairobi, Nairobi, Kenya; 6 Discipline of Public Health Medicine, School of Nursing and Public Health, University of KwaZulu-Natal, Durban, South Africa

**Keywords:** Cytokines, Infectious diseases, HIV infections

## Abstract

Previous studies have highlighted the role of pre-infection systemic inflammation on HIV acquisition risk, but the extent to which it predicts disease progression outcomes is less studied. Here we examined the relationship between pre-infection plasma cytokine expression and the rate of HIV disease progression in South African women who seroconverted during the CAPRISA 004 tenofovir gel trial. Bio-Plex 200 system was used to measure the expression of 47 cytokines/chemokines in 69 seroconvertors from the CAPRISA 004 trial. Cox proportional hazards regression analyses were used to measure associations between cytokine expression and CD4 decline prior to antiretroviral therapy initiation. Linear regression models were used to assess whether pre-infection cytokine expression were predictors of disease progression outcomes including peak and set-point viral load and CD4:CD8 ratio at less and greater than180 days post infection. Several cytokines were associated with increased peak HIV viral load (including IL-16, SCGFβ, MCP-3, IL-12p40, SCF, IFNα2 and IL-2). The strongest association with peak viral load was observed for SCGFβ, which was also inversely associated with lowest CD4:CD8 ratio < 180 days post infection and faster CD4 decline below 500 cells/µl (adjusted HR 4.537, 95% CI 1.475–13.954; *p* = 0.008) in multivariable analysis adjusting for age, study site, contraception, baseline HSV-2 status and trial arm allocation. Our results show that pre-infection systemic immune responses could play a role in HIV disease progression, especially in the early stages of infection.

## Introduction

Immune activation in response to HIV is critical in providing protective immunity to fight the infection, however those same responses can contribute to pathogenesis by providing the virus with increased supplies of activated target cells. Cytokine dysregulation and inflammation play major roles in HIV pathogenesis by contributing to CD4^+^ T cells depletion and increased viral load^1–3^. Immune activation was shown to be one of the major predictors of CD4^+^ T cell loss, independent of viral load^4^. The plasma cytokine storm during acute HIV infection predicts viral set-point and subsequent disease progression, with 66% of the variation in viral set-point 12 months post-infection predicted by plasma levels of 5 cytokines: interleukin (IL)-15, IL-7, IL-12p40, IL-12p70 and interferon gamma (IFNy)^5,6^. During chronic HIV infection, soluble plasma markers (such as IL-6, C-reactive protein and D-dimer) were shown to be predictive of comorbidities, disease progression and mortality in patients on and off antiretroviral therapy (ART)^7–10^.

While several studies have shown that systemic immunity and increased inflammation increase the risk of HIV acquisition^11–13^, few have looked at the effect of pre-infection systemic inflammation on HIV disease progression^14–16^. High CD4^+^ T cell counts prior to infection were associated with significantly higher rate of CD4^+^ T cell decline in the first 3 months of infection, likely related to increased HIV replication^16^. Understanding the impact of innate and adaptive immune function prior to HIV-1 infection on subsequent disease progression could contribute to the establishment of more optimized and personalized HIV treatment and prevention methods. In this study, we examined whether pre-infection plasma cytokine concentrations predicted the rate of HIV disease progression in the CAPRISA 004 cohort, a phase IIb randomized trial that evaluated the safety and effectiveness of 1% tenofovir gel for the prevention of HIV infection in South African women.

## Methods

### Study cohort

This study included women who HIV seroconverted while participating in CAPRISA 004, a phase IIb, randomised, double-blinded, placebo-controlled study of 1% tenofovir gel: “Safety and Effectiveness Study of a Candidate Vaginal Microbicide for Prevention of HIV” (NCT00441298)^17^. CAPRISA 004 enrolled sexually active, HIV uninfected females, aged between 18–40 years from rural (Vulindlela Research Clinic) and urban clinics (eThekwini Research Clinic) in KwaZulu Natal, South Africa from May 2007 to January 2009. Once seroconversion was confirmed, women were then followed up in the CAPRISA 002 Acute Infection cohort study^18^. This study was approved by the University of KwaZulu Natal Biomedical Research Ethics Committee (BREC/00001105/2020), and all participants provided the informed written consent prior to enrollment. All methods were carried out in accordance with relevant guidelines and regulations.

### Sample collection and processing

Peripheral blood was collected in acid citrate dextrose (ACD) tubes. Following centrifugation (10′, 1600 rpm) plasma was separated and cryopreserved at − 80 °C for further analysis.

### Soluble biomarker analysis

Cytokine/chemokine levels were measured using the Bio-Plex cytokine assays Group 1 (27 Plex) and Group II (21 Plex). Human cytokine 27-plex Assay (#M500KCAF0Y, BIO-RAD) included the following cytokines: IL-1β, IL-1rα, IL-2, IL-4, IL-5, IL-6, IL-7, IL-8, IL-9, IL-10, IL-12p70, IL-13, IL-15, IL-17α, Eotaxin, Basic FGF, G-CSF, GM-CSF, IFN-y, IP-10, MCP-1,MIP-1α, MIP-1β, PDGF-ββ, RANTES, TNF-α. Human 21-plex Assay (#MF0005KMII, BIO-RAD) included: IFN-α2, IL-1α, IL-2Rα, IL-3, IL-12p40, IL-16, CTACK, GROα, HGF, LIF, MCP-3, M-CSF, MIF, MIG, β-NGF, SCF, SCGF β, SDF-1α, TNF-β, and TRAIL. All assays were performed following manufacturer’s instructions. Samples with values below the lower detection limit were assigned the value half the lower limit of quantification, LLOQ/2.

### Statistical analysis

All cytokine concentrations were log_10_ transformed. Cytokines detectable at less than 60% frequency were analysed as binary variables (IL-1α and IL-2), and the remainder were analysed as continuous variables. Disease progression was evaluated using several readouts including: HIV viral load (VL), measured both as peak (highest value in the first 180 days of infection) and set point (average value in measurements made after 180 days of infection prior to ART initiation); and CD4:CD8 ratio, measured both as lowest CD4:CD8 ratio within the first 180 days post infection and the mean CD4:CD8 ratio > 180 days (average value in measurements made after 180 days of infection prior to ART initiation). Linear regression was used to assess the impact of each cytokine on VL and CD4:CD8 ratio in bivariate and multivariable models adjusting for age, contraceptive use, HSV-2 at baseline, study site and study arm at randomization. The rates of CD4 decline were compared in bivariate and multivariable Cox regression models with the endpoint defined as two consecutive CD4 counts below 500 cells/μl. Analysis excluded the CD4 counts during the first 180 days post infection due to transient CD4 decreases during acute infection, as previously described^19^. Multivariable analysis with and without the logVL as a predictor variable is reported for the CD4 decline analysis. Statistical significance was indicated as *p* value ≤ 0.05. Multiple comparisons adjustment was done using false discovery rate (FDR) method of Benjamini and Hochberg with FDR (Q) = 10%. All analyses were performed using SPSS v27 and GraphPad Prism Version 7.

## Results

### Study participants

We measured the expression of plasma cytokines from women who became HIV infected at three months after enrolment in the CAPRISA 004 study. A total of 69 women were sampled and 60 included in the final analysis. Plasma samples were obtained at a median of 330 days prior to infection (IQR 211–493). The median age was 22 (IQR 20–25), and the baseline HSV-2 seroprevalence was 63.2%. Most participants (73.9%) were using Depo-Provera (DMPA) as their preferred method of contraception. The demographic, behavioural and clinical variables are shown in Table 1.

**Table 1:** Study cohort characteristics.

Variables	Cases (n = 69)
Age, years [median (IQR)]	22 (20–25)
Sex acts in the last 30 days [median (IQR)]	6 (3–10)
Oldest sex partner, years [median (IQR)]	25 (23–28)
**Living with regular partner**	
No	62 (89.90%)
Yes	7 (10.10%)
**Highest level of education**	
Primary school not complete	3 (4.3%)
Primary Schooling complete	37 (53.6%)
High school complete	28 (40.6)
Tertiary education complete	1 (1.4%)
**Contraception**	
Depo-provera	51 (73.90%)
Hysterectomy	0
Nur-Isterate	13 (18.80%)
Oral	5 (7.20%)
Tubal ligation	0
**Marital status**	
Casual partner(s)	2 (2.90%)
Married	2 (2.90%)
Married and have casual partners	0
Stable partner(s)	61 (88.40%)
Stable and casual partners	4 (5.80%)
**Treatment arm (CAPRISA 004)**	
Placebo	42 (60.90%)
Tenofovir	27 (39.10%)
**Baseline HSV-2**	
No	25 (36.80%)
Yes	43 (63.20%)

### Pre-infection plasma cytokines/chemokines and viral load

**Figure 1:**
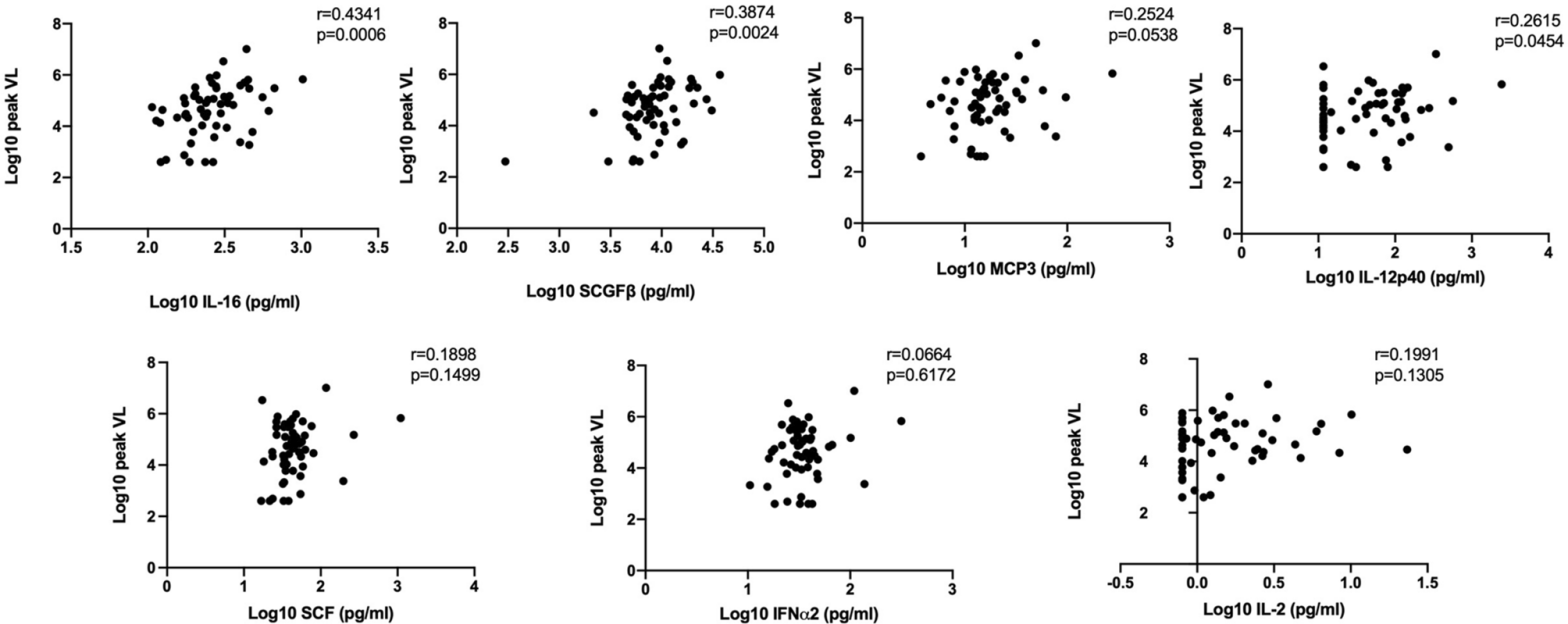
Correlation between plasma cytokines and peak VL (n = 60). Only significantly affected cytokines in multivariable linear regression analysis are shown in the figure. The r and *p* values depicted in the graphs are the result of the Spearman correlation analysis.

We performed bivariate and multivariable linear regression models to assess associations between pre-infection cytokines and HIV viral load. In bivariate analyses IL-16, serum stem cell growth factor beta (SCGFβ), monocyte chemotactic protein-3 (MCP-3), IL-12p40 and stem cell factor (SCF) concentrations were associated with increased peak viral load (Table 2). Similar results were obtained for these cytokines in models adjusted for contraceptive use, age, HSV-2 at baseline, study site and study arm [IL-16, adjusted(a)β = 2.065, 95% CI 0.786, 3.344, *p* = 0.002; SCGFβ, aβ = 1.345, 95% CI 0.536, 2.154, *p* = 0.002; MCP-3, aβ = 1.101, 95% CI 0.255,1.946, *p* = 0.012; IL-12p40, aβ = 0.643, 95% CI 0.126, 1.160, *p* = 0.016; SCF, aβ = 0.989, 95% CI 0.019, 1.959, *p* = 0.046; Table 2, Fig. 1]. Additionally, IFNα2 and IL-2 were significantly associated with peak viral load in multivariable analysis (IFNα2, aβ = 1.277, 95% CI, 0.088, 2.466, *p* = 0.036; IL-2, aβ = 0.835, 95% CI 0.011, 1.658, *p* = 0.047; Table 2, Fig. 1). We observed no significant association between pre-infection cytokines and set-point viral load (Sup. Table 2). It is likely that through their chemotactic and pro-inflammatory properties, persistent upregulation of the identified markers can lead to increased number of activated CD4^+^ T cells thereby fuelling early HIV replication.

**Table 2:** Bivariate and multivariable linear regression analysis on the effect of pre-infection plasma cytokines on peak viral load (*only statistically significant and trending cytokines/chemokines shown, for rest of the data see Sup. Table 1).

Variables	Bivariate				Multivariable			
	Β-coefficient	95% CI		*p* Value	aβ-coefficient	95% CI		*p* Value
		Lower	Upper			Lower	Upper	
Log IL-16	1.939	0.732	3.146	0.002*	2.065	0.786	3.344	0.002*
Log SCGFβ	1.310	0.517	2.104	0.002*	1.345	0.536	2.154	0.002*
Log MCP-3	0.858	0.063	1.653	**0.035**	1.101	0.255	1.946	**0.012**
Log IL-12p40	0.500	0.031	0.969	**0.037**	0.643	0.126	1.160	**0.016**
Log SCF	0.950	0.039	1.861	**0.041**	0.989	0.019	1.959	**0.046**
Log IFNα2	0.975	− 0.145	2.096	0.087	1.277	0.088	2.466	**0.036**
Log IL-2	0.679	− 0.096	1.454	0.085	0.835	0.011	1.658	**0.047**

Significant values are in bold.

Dependent variable: Log10 viral load; both models included study arm; multivariable analysis adjusted for contraception, age, study site, HSV-2 at baseline and study arm.

*p* Values that passed the FDR correction are indicated with a *.

### Pre-infection plasma cytokines/chemokines and CD4:CD8 ratio post-infection

**Figure 2:**
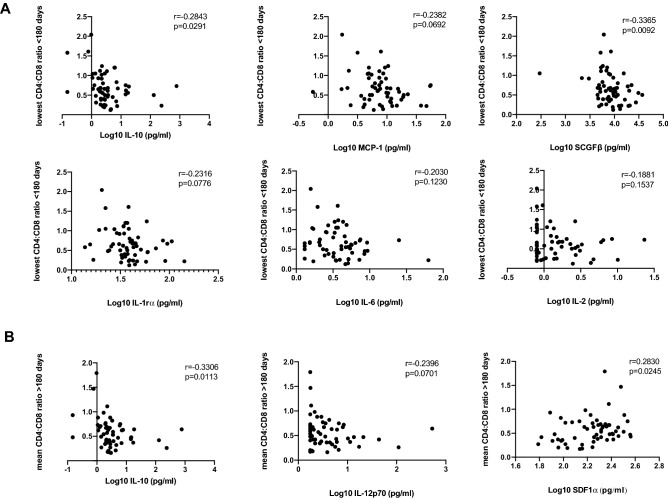
Correlation between plasma cytokines and lowest CD4:CD8 ratio < 180 days post infection (**A**) and mean CD4:CD8 ratio > 180 days post infection (**B**) (n = 60). Only significantly affected cytokines in multivariable linear regression analysis are shown in the figure. The r and p values depicted in the graphs are the result of the Spearman correlation analysis.

Next we used linear regression models to evaluate if pre-infection cytokine expression was associated with lowest CD4:CD8 ratio < 180 days post infection. Six cytokines were negatively associated with lowest CD4:CD8 ratio in the bivariate model: IL-10, MCP-1, SCGFβ, IL-1rα, IL-6 and IL-2 (Table 3). Similar results were obtained following multivariable analysis: IL-10: aβ =  − 0.216, 95% CI (− 0.369, − 0.062), *p* = 0.007; MCP-1: aβ =  − 0.273; 95% CI (− 0.530, − 0.017), *p* = 0.037; SCGFβ: aβ =  − 0.344, 95% CI (− 0.644, − 0.043), *p* = 0.026; IL-1rα: aβ =  − 0.551, 95% CI (− 1.021, − 0.082), *p* = 0.022; IL-6: aβ =  − 0.361, 95% CI (− 0.662, − 0.059), *p* = 0.020; IL-2: aβ =  − 0.347, 95% CI (− 0.635, − 0.060), *p* = 0.019; Table 3, Fig. 2A. As SCGFβ was also associated with peak viral load, its effect on decreased CD4:CD8 ratio is likely mediated through increase in HIV replication and associated CD4^+^ T cell death, while the effect of the other cytokines could be mediated through immune activation/exhaustion. Fewer associations with CD4:CD8 ratio > 180 days post infection were observed, with IL-10 and stromal cell-derived factor-1 alpha (SDF-1α) being significant in bivariate analysis (Table 4). IL-10 remained significantly associated after adjusting for contraception, age, study site, study arm, age and HSV-2 serostatus at baseline [aβ =  − 0.153, 95% CI (− 0.282, − 0.024), *p* = 0.021] Table 4, Fig. 2B. Additionally, increased IL-12p70 was significantly associated with lower mean CD4:CD8 ratios > 180 in the multivariable analysis [aβ =  − 0.202, 95%CI (− 0.376, − 0.029), *p* = 0.023] Table 4, Fig. 2B.

**Table 3:** Bivariate and multivariable linear regression analysis on the effect of pre-infection plasma cytokines on minimum CD4:CD8 ratio < 180 post infection (*only statistically significant and trending cytokines/chemokines shown, for rest of the data see Sup. Table 3).

Variables	Bivariate				Multivariable			
	β-coefficient	95% CI		*p* Value	aβ-coefficient	95% CI		*p* Value
		Lower	Upper			Lower	Upper	
Log IL-10	− 0.206	− 0.353	− 0.060	**0.007**	− 0.216	− 0.369	− 0.062	**0.007**
Log MCP-1	− 0.284	− 0.533	− 0.035	**0.026**	− 0.273	− 0.530	− 0.017	**0.037**
Log SCGFβ	− 0.373	− 0.663	− 0.083	**0.013**	− 0.344	− 0.644	− 0.043	**0.026**
Log IL-1rα	− 0.571	− 1.027	− 1.114	**0.015**	− 0.551	− 1.021	− 0.082	**0.022**
Log IL-6	− 0.351	− 0.645	− 0.057	**0.020**	− 0.361	− 0.662	− 0.059	**0.020**
Log IL-2	− 0.329	− 0.597	− 0.061	**0.017**	− 0.347	− 0.635	− 0.060	**0.019**

Significant values are in bold.

Dependent variable: CD4:CD8 ratio; both models included study arm; multivariable analysis adjusted for contraception, age, study site, HSV-2 at baseline and study arm.

**Table 4:** Bivariate and multivariable linear regression analysis on the effect of pre-infection plasma cytokines on mean CD4:CD8 ratio > 180 post infection (*only significant and trending cytokines/chemokines shown, for rest of the data see Sup. Table 4).

Variables	Bivariate				Multivariable			
	β-coefficient	95% CI		*p* Value	aβ-coefficient	95% CI		*p* Value
		Lower	Upper			Lower	Upper	
Log IL-10	− 0.135	− 0.262	− 0.008	**0.038**	− 0.153	− 0.282	− 0.024	**0.021**
Log IL-12p70	− 0.130	− 0.295	0.035	0.120	− 0.202	− 0.376	− 0.029	**0.023**
Log SDF-1α	0.430	0.047	0.814	**0.029**	0.248	− 0.297	0.793	0.365

Significant values are in bold.

Dependent variable: CD4:CD8 ratio; both models included study arm; multivariable analysis adjusted for contraception, age, study site, HSV-2 at baseline and study arm.

### Pre-infection plasma cytokine expression and CD4 decline

We next performed bivariate and multivariable Cox regression analyses of pre-infection plasma cytokines and time to CD4 decline below 500 cells/μl prior to ART initiation. Pre-infection plasma SCGFβ (HR = 3.71, 95% CI 1.28–10.77, *p* = 0.016) and TNFβ (HR = 2.24, 95% CI 1.13–4.45, *p* = 0.022) levels significantly predicted faster CD4 loss in the bivariate analysis (Table 5). Additionally, there was a trend for IL-16 (HR = 4.546, 95% CI 0.919–22.484, *p* = 0.063, Table 5). In the multivariable model adjusting for contraception, age, study site, study arm, VL and HSV-2 at baseline IL-2 (aHR = 0.318, 95% CI 0.109–0.926, *p* = 0.036), growth-regulated oncogene alpha (GROα) (aHR = 0.13, 95% CI 0.018–0.965, *p* = 0.046), IFNα2 (aHR = 0.118, 95% CI 0.016–0.847, *p* = 0.034) and SDF-1α (aHR = 0.087, 95% CI 0.009–0.853, *p* = 0.036) correlated with slower CD4 decline (Sup. Table 5). This model corrected for HIV viral load indicating that these cytokines likely affect CD4 decline in viral load-independent mechanisms, most likely due to increased T cell function and upregulation of anti-viral immune responses. We next ran a multivariable model excluding viral load, as seen in the bivariate model, SCGFβ (aHR = 4.537, 95% CI 1.475–13.954, *p* = 0.008) and TNFβ (aHR = 2.318, 95% CI 1.133–4.741, *p* = 0.021 (Table 5) were associated with faster CD4 decline suggesting that this effect may be due to increases in viral replication, in addition to increased immune activation. We additionally observed a trend for IL-16 (aHR = 4.595, 95% CI 0.907–23.270, *p* = 0.065) and MIP1β (aHR = 4.079, 95% CI 0.957–17.391, *p* = 0.057) being associated with faster CD4 decline.

**Table 5:** Bivariable and multivariable survival analysis of the effect of pre-infection plasma cytokines on CD4 decline < 500 µl (*only statistically significant and trending cytokines/chemokines shown, for rest of the data see Sup. Table 5).

Variables	HR (95% CI)	*p* Value	aHR *(95% CI)	*p* Value	aHR (-VL)*(95% CI)	*p* Value
log SCGFβ	3.712 (1.279–10.771)	**0.016**	2.738 (0.729–10.290)	0.136	4.537(1.475–13.954)	**0.008**
log TNFβ	2.236 (1.125–4.445)	**0.022**	1.745 (0.813–3.746)	0.153	2.318 (1.133–4.741)	0.002*
log IL-16	4.546 (0.919–22.484)	0.063	0.988 (0.154–6.326	0.990	4.595 (0.907–23.270)	0.065
log IL-2	0.936 (0.418–2.094)	0.871	0.318 (0.109–0.926)	**0.036**	0.905 (0.368–2.225)	0.828
log GROα	0.387 (0.081–1.846)	0.234	0.131 (0.018–0.965)	**0.046**	0.416 (0.084–2.051)	0.281
log IFNα2	1.208 (0.232–6.287)	0.822	0.118 (0.016–0.847)	**0.034**	1.663 (0.277–9.970)	0.578
log SDF-1α	0.666 (0.159–2.792)	0.578	0.087 (0.009–0.853)	**0.036**	1.082 (0.145–8.076)	0.939
log IL-10	1.109 (0.743–1.656)	0.613	0.592 (0.349–1.005)	0.052	1.046 (0.657–1.666)	0.850
log IL-3	0.866 (0.248–3.022)	0.822	0.268 (0.069–1.039)	0.057	1.083 (0.269–4.359)	0.910
log TRAIL	0.728 (0.303–1.751)	0.479	0.446 (0.196–1.015)	0.054	0.641 (0.247–1.660)	0.360
log MIP-1β	2.369 (0.630–8.911)	0.202	3.882 (0.675–22.339)	0.129	4.079 (0.957–17.391)	0.057

Significant values are in bold.

Study arm was included in all models; multivariable analysis adjusted for contraception, age, study site, HSV-2 at baseline study arm, + / − VL.

*p* Values that passed the FDR correction are indicated with a *.

## Discussion

Systemic inflammation is considered to be a driving force underlying CD4^+^ T cell depletion and immune dysfunction during HIV disease progression, and it is known to persist in ARV-treated individuals with suppressed viral load^20^. Numerous studies have investigated the effects of systemic inflammation during the acute and chronic infection; however, few have assessed the impact of pre-infection immune status on disease progression. Pre-infection levels of cytokines likely reflect the immunological status of the individual and could therefore be important in assessing the risk of early stage disease progression. Here we characterised the impact of pre-infection plasma cytokines on the markers of HIV disease progression including viral load, CD4:CD8 ratio and CD4 decline.

We found that expression of five cytokines, IL-16, SCGFβ, MCP-3, IL-12p40, and SCF had an impact on the peak viral load following bivariate and multivariable linear regression analyses. The observed effect was strongest for IL-16 and SCGFβ. IL-16, originally named as lymphocyte chemoattractant factor (LCF), is produced by a range of cell types including activated CD8^+^ T cells, macrophages and dendritic, and it is a potent chemoattractant for all peripheral immune cells expressing CD4 receptor^21^. IL-16 was shown to prevent HIV replication ex vivo*,* with the inhibitory effect likely mediated at the level of transcriptional regulation^21–25^. While IL-16 can likely contribute to hindrance of HIV replication at the site of exposure, once infection is established increased systemic levels of IL-16, can contribute to increased CD4^+^ T cell recruitment during acute infection thereby increasing HIV replication and subsequent disease progression. SCGFβ is a growth factor for hematopoietic progenitor cells (HPCs) and has chemotactic properties^26^. While there is a lack of data on the role of the SCGFβ in inflammation and HIV infection, elevated plasma levels of SCGFβ could potentially induce proliferation and activation of HIV target cells, thereby contributing to the increased viral load during early infection. During early infection, MCP-3 and IL-12p40, through their pro-inflammatory properties and as a cause and consequence of macrophage/dendritic cell recruitment, can lead to increased HIV replication^27–31^. We also found IFN-α2 to be significantly associated with peak-viral load. Continuous production of IFN-α has been associated with rapid HIV-disease progression, based on the likely effect of IFN-α to induce T cell differentiation and activation^32^. It has also been shown that transmitted founder viruses are most likely to replicate and spread in the presence of IFN-α^33^. SCF and IL-2 promote differentiation and proliferation of HIV target cells^34^ and could contribute to increase in the availability of cells susceptible to HIV infection, thereby resulting in increased viral replication. We observed no significant associations between pre-infection soluble cytokine/chemokine expression and set point VL, suggesting that pre-infection immune status likely has a greater impact on early stages of HIV infection. HIV viral load is likely determined by other factors including CD8 responses after infection is established^35^.

In addition to viral load, CD4:CD8 ratio is another commonly used marker of HIV disease progression and HIV-linked immune dysregulation^36^. HIV is associated with the reduction of CD4^+^ T cells and the increase of activated cytotoxic T lymphocytes specific to HIV^37^. Here we found that increased plasma concentrations of IL-10, MCP-1, IL-1rα, IL-2, IL-6 and SCGFβ had a negative impact on CD4:CD8 ratio during acute infection (< 180 days post infection), as evaluated by linear regression model. IL-10 is part of the broad “cytokine storm” in acute HIV infection and limits the magnitude and the functional capacity of effector CD4^+^ T cells^38–40^. IL-1rα is an inflammatory cytokine that is rapidly produced by numerous cells in response to infection and modulates IL-1 activity^41^. Increased IL-1rα and IL10 expression prior to infection can compromise early immune response to HIV and result in reduced CD4:CD8 ratio. MCP1, SCGFβ and IL-6 effect is likely mediated through its chemotactic and pro-inflammatory properties^26,42^ and CD4^+^ T cell polarization towards a Th2 phenotype^43^. Furthermore, IL-10, IL-12p70 and SDF-1α were found to be significantly associated with lower CD4:CD8 ratios during chronic phase (> 180 days post infection). Since IL-10 is involved in lessening the effector CD4^+^ T cell responses, increased pre-infection levels of this cytokine can potentially downregulate immune responses responsible for combating HIV. Increased pre-infection inflammation likely has a more profound effect during acute infection through HIV target cell recruitment and activation, while effects on later stages of infection are mediated through impairment and downregulation of host immune responses.

We also examined the effect of pre-infection plasma cytokine levels on CD4^+^ T cell decline below 500 μl prior to ART initiation. Increased understanding of the causes of CD4 decline in HIV patients is important for clinical management of the disease^44^. In our study, 2 of the pre-infection plasma cytokines (SCGFβ and TNFβ) significantly predicted faster CD4^+^ T loss. Cytokines that were associated with slower CD4 decline in the multivariable model adjusting for VL included IL-2, GROα, IFNα2, SDF-1α. Since VL is one of the main drivers of CD4 decline during HIV infection, in order to determine if the effect of cytokines identified in the bivariate analysis was mainly driven by VL, we re-ran the multivariable model excluding correction for VL measure. In the multivariable model excluding VL, following cytokines were significantly associated with CD4 decline: MIP1β, IL-16, SCGFβ and TNFβ indicating that their effect on CD4 decline is at least partly mediated through HIV replication and the associated VL changes. This is supported by the results looking at the peak VL, where both IL-16 and SCGFβ were associated with increased peak VL. The effect of pre-infection IL-2, GROα, IFNα2, and SDF1-α on CD4 decline is likely to be independent of VL and HIV replication and instead may be reflective of pre-infection immune state that is favourable in the context of HIV disease progression once the person is infected.

Our study has several limitations. Our findings are specific to young women in South Africa and should be validated in other settings. We cannot exclude the effect of other unmeasured co-infections on the inflammatory signatures in the studied cohort. Additional limitation of this study includes the fact that the cytokine measurements were not made at the last HIV-negative visit, closest to the infection time. However, despite this, our study shows that pre-infection systemic cytokine/chemokine expression seems to play a significant role in determining the rate of disease progression. Once a person is infected, level of existing systemic inflammation can determine the amount of activated CD4^+^ T cells that are available to the virus; increased levels of activated CD4^+^ T cells can result in increased peak viral load and a more profound drop in CD4^+^ T cell numbers ultimately leading to worsened disease progression outcome.

## Supplementary Information

Supplementary Information.
