# Supplementary material for: Pre-infection plasma cytokines and chemokines as predictors of HIV disease progression
Source: Sci Rep. 2022 Feb 14;12:2437. doi: 10.1038/s41598-022-06532-w (PMC8844050; doi:10.1038/s41598-022-06532-w)
Supplement: Supplementary file 1 — Supplementary Information. [file 41598_2022_6532_MOESM1_ESM.pdf]

Supplementary materials:

Sup. Table 1. Bivariate and multivariable linear regression analysis on the effect of pre-infection plasma cytokines on peak viral load

	Bivariable				Multivariable			
			95.0% CI for B				95.0% CI for B	
	**β-coefficient**	**Sig.**	**Lower Bound**	**Upper Bound**	**aβ-coefficient**	**Sig.**	**Lower Bound**	**Upper Bound**
logIL1β	-0.009	0.986	-1.040	1.021	0.004	0.995	-1.050	1.057
logIL1ra	0.593	0.382	-0.756	1.941	0.599	0.389	-0.783	1.981
logIL2	0.679	0.085	-0.096	1.454	0.835	**0.047**	0.011	1.658
logIL4	-0.145	0.785	-1.206	0.915	-0.152	0.778	-1.228	0.924
logIL5	0.147	0.639	-0.477	0.772	0.197	0.534	-0.434	0.828
logIL6	0.348	0.424	-0.518	1.214	0.435	0.330	-0.453	1.324
logIL7	-0.094	0.809	-0.875	0.686	-0.026	0.951	-0.857	0.805
logIL8	0.281	0.676	-1.058	1.620	0.338	0.628	-1.054	1.730
logIL9	-0.043	0.913	-0.824	0.738	-0.082	0.840	-0.896	0.732
logIL10	0,276	0,209	-0.160	0.712	0.331	0.151	-0.125	0.787
logIL12p70	-0.097	0.731	-0.659	0.465	-0.031	0.922	-0.654	0.593
logIL13	0.182	0.635	-0.582	0.946	0.189	0.634	-0.602	0.979
logIL15	-0.083	0.822	-0.822	0.655	-0.087	0.819	-0.849	0.674
logIL17	0.097	0.707	-0.419	0.613	0.037	0.893	-0.515	0.589
logEotaxin	-0.144	0.754	-1.058	0.771	0.022	0.964	-0.934	0.978
logFGFbasic	-0.232	0.374	-0.751	0.287	-0.294	0.298	-0.854	0.267
logGCSF	-0.417	0.394	-1.389	0.555	-0.284	0.571	-1.282	0.715
logGMCSF	-0.150	0.629	-0.767	0.467	-0.266	0.421	-0.924	0.392
logIFNg	0.091	0.765	-0.516	0.698	0.153	0.623	-0.466	0.771
logIP10	-0.505	0.148	-1.193	0.184	-0.474	0.265	-1.319	0.370
logMCP1	0.202	0.582	-0.530	0.934	0.206	0.585	-0.546	0.957
logMIP1a	-0.290	0.603	-1.402	0.822	-0.123	0.835	-1.302	1.056
logPDGFbb	-0.269	0.300	-0.784	0.246	-0.333	0.225	-0.879	0.212
logMIP1b	0.421	0.490	-0.792	1.634	0.683	0.287	-0.591	1.958
logRANTES	-0.414	0.422	-1.438	0.611	-0.482	0.366	-1.545	0.580
logTNFa	-0.276	0.483	-1.060	0.507	-0.189	0.643	-1.000	0.623

logVEGF	0.091	0.863	-0.969	1.152	0.132	0.813	-0.983	1.247
logIL1a	0.790	0.098	-0.150	1.729	0.714	0.158	-0.287	1.715
logIL2Ra	0.082	0.866	-0.896	1.061	0.226	0.672	-0.838	1.290
logIL3	0.333	0.382	-0.425	1.091	0.513	0.214	-0.304	1.330
logIL12p40	0.500	**0.037**	0.031	0.969	0.643	**0.016**	0.126	1.160
logIL16	1.939	**0.002**	0.732	3.146	2.065	**0.002**	0.786	3.344
logCTACK	0.429	0.097	-0.080	0.939	0.489	0.066	-0.033	1.012
logGROa	0.548	0.326	-0.560	1.655	0.518	0.366	-0.621	1.656
logHGF	0.821	0.067	-0.058	1.701	0.773	0.116	-0.199	1.744
logIFNa2	0.975	0.087	-0.145	2.096	1.277	**0.036**	0.088	2.466
logLIF	0.532	0.114	-0.132	1.195	0.603	0.088	-0.093	1.299
logMCP3	0.858	**0.035**	0.063	1.653	1.101	**0.012**	0.255	1.946
logMCSF	0.330	0.306	-0.310	0.969	0.309	0.388	-0.404	1.022
logMIF	0.132	0.759	-0.722	0.985	0.071	0.874	-0.826	0.969
logMIG	0.522	0.151	-0.196	1.241	0.507	0.175	-0.233	1.248
logNGF	0.168	0.686	-0.661	0.996	0.295	0.510	-0.597	1.187
logSCF	0.950	**0.041**	0.039	1.861	0.989	**0.046**	0.019	1.959
logSCGFb	1.310	**0.002**	0.517	2.104	1.345	**0.002**	0.536	2.154
logSDF1a	0.327	0.616	-0.974	1.629	1.339	0.117	-0.348	3.026
logTNFb	0.428	0.108	-0.097	0.952	0.421	0.140	-0.143	0.986
logTRAIL	0.106	0.733	-0.513	0.725	0.008	0.980	-0.658	0.675

- Dependent variable: Log10 viral load; both models included study arm; multivariable analysis adjusted for contraception, age, study site, HSV-2 at baseline and study arm.
- p-values that passed the FDR correction are indicated with a *

Sup. Table 2. Bivariate and multivariable linear regression analysis on the effect of pre-infection plasma cytokines on the setpoint viral load

	Bivariable				Multivariable			
			95.0% CI for B				95.0% CI for B	
	β-coefficient	Sig.	Lower Bound	Upper Bound	aβ-coefficient	Sig.	Lower Bound	Upper Bound
log_IL1b	-0.254	0.575	-1.158	0.650	-0.099	0.826	-0.994	0.796
log_IL1ra	0.016	0.977	-1.109	1.142	0.104	0.852	-1.008	1.216
log_IL2	-0.124	0.699	-0.766	0.517	-0.065	0.846	-0.737	0.606
log_IL4	-0.196	0.670	-1.111	0.720	-0.205	0.651	-1.106	0.697
log_IL5	0.104	0.682	-0.402	0.611	0.174	0.488	-0.326	0.674
log_IL6	-0.288	0.460	-1.064	0.488	-0.111	0.777	-0.890	0.668
log_IL7	-0.068	0.828	-0.692	0.556	0.049	0.881	-0.605	0.704
log_IL8	0.136	0.806	-0.970	1.243	0.233	0.676	-0.878	1.344
log_IL9	-0.127	0.697	-0.777	0.523	-0.188	0.567	-0.844	0.468
log_IL10	0.123	0.467	-0.213	0.459	0.167	0.331	-0.174	0.508
log_IL12p70	-0.223	0.299	-0.649	0.203	-0.106	0.645	-0.568	0.355
log_IL13	-0.024	0.941	-0.656	0.609	0.002	0.994	-0.642	0.647
log_IL15	0.126	0.659	-0.442	0.693	0.125	0.662	-0.444	0.693
log_IL17	0.245	0.264	-0.190	0.679	0.128	0.577	-0.330	0.585
log_Eotaxin	-0.530	0.131	-1.222	0.163	-0.336	0.350	-1.050	0.379
log_FGFbasic	0.126	0.546	-0.290	0.542	0.073	0.741	-0.366	0.511
log_GCSF	-0.489	0.258	-1.346	0.368	-0.263	0.550	-1.139	0.614
log_GMCSF	-0.113	0.652	-0.611	0.386	-0.265	0.303	-0.776	0.246
log_IFNg	0.029	0.908	-0.472	0.530	0.110	0.657	-0.386	0.606
log_IP10	-0.483	0.067	-1.002	0.036	-0.321	0.307	-0.947	0.304
log_MCP1	0.021	0.942	-0.563	0.605	0.079	0.786	-0.499	0.657
log_MIP1a	-0.787	0.114	-1.768	0.194	-0.495	0.354	-1.557	0.567
log_PDGFbb	0.106	0.598	-0.294	0.505	0.002	0.990	-0.411	0.416
log_MIP1b	-0.247	0.602	-1.191	0.698	-0.022	0.964	-0.995	0.950
log_RANTES	0.281	0.479	-0.508	1.069	0.181	0.649	-0.615	0.978
log_TNFa	-0.196	0.546	-0.841	0.450	-0.054	0.869	-0.705	0.597
log_VEGF	0.013	0.980	-1.009	1.035	0.304	0.560	-0.737	1.346
log_IL1a	1.815	0.093	-0.314	3.944	2.035	0.072	-0.186	4.256

log_IL2Ra	-0.795	0.109	-1.774	0.184	-0.396	0.485	-1.527	0.734
log_IL3	-0.378	0.292	-1.091	0.335	-0.034	0.932	-0.838	0.769
log_IL12p40	-0.068	0.742	-0.477	0.342	0.095	0.676	-0.358	0.548
log_IL16	0.718	0.186	-0.356	1.792	0.842	0.134	-0.269	1.953
log_CTACK	-0.109	0.589	-0.509	0.292	-0.049	0.808	-0.455	0.357
log_GROa	0.325	0.559	-0.784	1.434	0.480	0.391	-0.634	1.594
log_HGF	0.192	0.645	-0.639	1.024	0.076	0.865	-0.819	0.971
log_IFNa2	0.063	0.905	-0.986	1.111	0.616	0.280	-0.516	1.748
log_LIF	-0.014	0.969	-0.744	0.715	0.270	0.480	-0.491	1.030
log_MCP3	0.308	0.395	-0.412	1.029	0.651	0.097	-0.121	1.424
log_MCSF	0.113	0.734	-0.552	0.779	0.173	0.632	-0.548	0.894
log_MIF	-0.054	0.887	-0.812	0.703	-0.107	0.782	-0.877	0.664
log_MIG	-0.186	0.522	-0.764	0.392	-0.148	0.612	-0.728	0.433
log_NGF	-0.438	0.298	-1.272	0.397	-0.037	0.938	-0.996	0.921
log_SCF	-0.286	0.547	-1.230	0.659	-0.143	0.773	-1.131	0.845
log_SCGFb	0.524	0.116	-0.133	1.181	0.502	0.133	-0.158	1.161
log_SDF1a	-0.529	0.305	-1.552	0.494	0.390	0.581	-1.021	1.802
log_TNFb	0.199	0.434	-0.307	0.705	0.287	0.274	-0.234	0.807
log_TRAIL	-0.279	0.274	-0.785	0.227	-0.326	0.214	-0.847	0.195

- Dependent variable: Log10 viral load; both models included study arm; multivariable analysis adjusted for contraception, age, study site, HSV-2 at baseline and study arm.

Sup. Table 3 Bivariate and multivariable linear regression analysis on the effect of pre-infection plasma cytokines on the minimum CD4:CD8 ratio < 180 post infection

	**Bivariable**				**Multivariable**			
			**95.0% CI for B**				**95.0% Confidence Interval for B**	
	**β-coefficient**	**Sig.**	**Lower Bound**	**Upper Bound**	**aβ-coefficient**	**Sig.**	**Lower Bound**	**Upper Bound**
log_IL1b	-0.245	0.178	-0.604	0.115	-0.248	0.181	-0.616	0.119
log_IL1ra	-0.571	**0.015**	-1.027	-0.114	-0.551	**0.022**	-1.021	-0.082
log_IL2	-0.329	**0.017**	-0.597	-0.061	-0.347	**0.019**	-0.635	-0.060
log_IL4	-0.163	0.385	-0.537	0.210	-0.134	0.481	-0.515	0.246
log_IL5	-0.053	0.631	-0.275	0.168	-0.068	0.545	-0.292	0.156
log_IL6	-0.351	**0.020**	-0.645	-0.057	-0.361	**0.020**	-0.662	-0.059
log_IL7	-0.005	0.969	-0.282	0.271	-0.050	0.734	-0.345	0.244
log_IL8	-0.400	0.089	-0.863	0.064	-0.366	0.136	-0.850	0.119
log_IL9	-0.204	0.138	-0.475	0.068	-0.174	0.227	-0.458	0.111
log_IL10	-0.206	**0.007**	-0.353	-0.060	-0.216	**0.007**	-0.369	-0.062
log_IL12p70	-0.154	0.118	-0.349	0.040	-0.196	0.072	-0.411	0.018
log_IL13	-0.148	0.274	-0.417	0.121	-0.181	0.194	-0.458	0.095
log_IL15	-0.072	0.582	-0.334	0.189	-0.088	0.517	-0.357	0.182
log_IL17	-0.151	0.095	-0.330	0.027	-0.116	0.234	-0.309	0.077
log_Eotaxin	-0.031	0.851	-0.355	0.294	-0.112	0.508	-0.450	0.226
log_FGFbasic	-0.032	0.729	-0.217	0.153	0.016	0.876	-0.185	0.217
log_GCSF	-0.143	0.409	-0.488	0.202	-0.183	0.302	-0.534	0.169
log_GMCSF	-0.087	0.427	-0.305	0.131	-0.024	0.835	-0.259	0.210
log_IFNg	-0.171	0.110	-0.381	0.040	-0.173	0.112	-0.388	0.042
log_IP10	0.050	0.690	-0.199	0.298	-0.062	0.682	-0.365	0.241
log_MCP1	-0.284	**0.026**	-0.533	-0.035	-0.273	**0.037**	-0.530	-0.017
log_MIP1a	-0.025	0.901	-0.420	0.370	-0.096	0.645	-0.514	0.321
log_PDGFbb	-0.046	0.616	-0.230	0.138	-0.009	0.930	-0.205	0.188
log_MIP1b	-0.157	0.469	-0.586	0.273	-0.174	0.445	-0.629	0.280
log_RANTES	-0.037	0.840	-0.402	0.328	0.033	0.862	-0.347	0.413
log_TNFa	-0.140	0.313	-0.417	0.136	-0.145	0.314	-0.431	0.141
log_VEGF	-0.170	0.364	-0.543	0.203	-0.170	0.391	-0.563	0.223
log_IL1a	-0.189	0.266	-0.526	0.148	-0.144	0.424	-0.503	0.215

log_IL2Ra	-0.113	0.516	-0.457	0.232	-0.181	0.335	-0.555	0.192
log_IL3	-0.119	0.378	-0.387	0.149	-0.193	0.185	-0.482	0.096
log_IL12p40	-0.081	0.348	-0.252	0.090	-0.123	0.200	-0.314	0.067
log_IL16	-0.259	0.264	-0.719	0.201	-0.249	0.315	-0.740	0.243
log_CTACK	-0.100	0.278	-0.283	0.083	-0.112	0.237	-0.301	0.076
log_GROa	-0.174	0.379	-0.566	0.219	-0.188	0.352	-0.591	0.214
log_HGF	-0.251	0.115	-0.565	0.063	-0.217	0.215	-0.563	0.130
log_IFNa2	-0.263	0.195	-0.664	0.138	-0.372	0.086	-0.799	0.054
log_LIF	-0.154	0.197	-0.391	0.082	-0.176	0.162	-0.424	0.073
log_MCP3	-0.207	0.156	-0.494	0.081	-0.268	0.088	-0.578	0.041
log_MCSF	-0.196	0.082	-0.419	0.026	-0.174	0.167	-0.424	0.075
log_MIF	-0.238	0.113	-0.533	0.058	-0.188	0.234	-0.501	0.125
log_MIG	-0.184	0.153	-0.438	0.070	-0.192	0.147	-0.453	0.070
log_NGF	-0.113	0.442	-0.405	0.179	-0.197	0.211	-0.509	0.115
log_SCF	-0.271	0.102	-0.598	0.056	-0.285	0.106	-0.633	0.062
log_SCGFb	-0.373	**0.013**	-0.663	-0.083	-0.344	**0.026**	-0.644	-0.043
log_SDF1a	0.115	0.620	-0.346	0.575	-0.194	0.526	-0.803	0.415
log_TNFb	-0.172	0.067	-0.356	0.013	-0.174	0.085	-0.372	0.025
log_TRAIL	-0.119	0.277	-0.336	0.098	-0.087	0.459	-0.322	0.147

- Dependent variable: CD4:CD8 ratio; both models included study arm; multivariable analysis adjusted for contraception, age, study site, HSV-2 at baseline and study arm.
- p-values that passed the FDR correction are indicated with a *

Sup. Table 4. Bivariate and multivariable linear regression analysis on the effect of pre-infection plasma cytokines on mean CD4:CD8 ratio >180 post infection

	Bivariable				Multivariable			
			95.0% CI for B				95.0% CI for B	
	β-coefficient	Sig.	Lower Bound	Upper Bound	aβ-coefficient	Sig.	Lower Bound	Upper Bound
log_IL1b	-0.164	0.355	-0.516	0.188	-0.212	0.227	-0.561	0.136
log_IL1ra	-0.227	0.301	-0.664	0.209	-0.239	0.274	-0.673	0.195
log_IL2	-0.128	0.309	-0.377	0.121	-0.162	0.220	-0.423	0.100
log_IL4	-0.066	0.713	-0.425	0.292	-0.058	0.746	-0.414	0.299
log_IL5	-0.005	0.964	-0.203	0.194	-0.036	0.713	-0.234	0.162
log_IL6	-0.176	0.246	-0.478	0.125	-0.220	0.149	-0.522	0.082
log_IL7	0.032	0.793	-0.212	0.276	-0.054	0.674	-0.313	0.204
log_IL8	-0.171	0.431	-0.602	0.261	-0.165	0.453	-0.602	0.272
log_IL9	-0.133	0.294	-0.386	0.119	-0.121	0.349	-0.379	0.136
log_IL10	-0.135	**0.038**	-0.262	-0.008	-0.153	**0.021**	-0.282	-0.024
log_IL12p70	-0.130	0.120	-0.295	0.035	-0.202	**0.023**	-0.376	-0.029
log_IL13	-0.049	0.694	-0.296	0.199	-0.114	0.371	-0.366	0.139
log_IL15	0.000	0.998	-0.223	0.222	-0.028	0.804	-0.253	0.197
log_IL17	-0.143	0.092	-0.311	0.024	-0.105	0.246	-0.283	0.074
log_Eotaxin	0.061	0.659	-0.215	0.338	-0.033	0.814	-0.318	0.251
log_FGFbasic	0.020	0.811	-0.144	0.183	0.066	0.446	-0.107	0.239
log_GCSF	-0.092	0.587	-0.431	0.246	-0.195	0.260	-0.538	0.148
log_GMCSF	-0.030	0.762	-0.225	0.166	0.045	0.661	-0.159	0.248
log_IFNg	-0.119	0.224	-0.312	0.075	-0.136	0.163	-0.328	0.057
log_IP10	0.100	0.340	-0.108	0.307	-0.041	0.743	-0.290	0.208
log_MCP1	-0.201	0.076	-0.423	0.022	-0.199	0.077	-0.421	0.022
log_MIP1a	0.147	0.456	-0.244	0.537	-0.026	0.902	-0.449	0.397
log_PDGFbb	-0.023	0.774	-0.179	0.134	0.025	0.755	-0.138	0.189
log_MIP1b	0.091	0.624	-0.279	0.461	0.051	0.792	-0.333	0.435
log_RANTES	-0.133	0.392	-0.441	0.176	-0.070	0.659	-0.384	0.245
log_TNFa	-0.092	0.470	-0.344	0.161	-0.121	0.346	-0.376	0.134
log_VEGF	-0.158	0.431	-0.555	0.240	-0.250	0.223	-0.657	0.157
log_IL1a	-0.281	0.508	-1.125	0.564	-0.177	0.691	-1.066	0.712
log_IL2Ra	0.052	0.791	-0.337	0.440	-0.111	0.614	-0.551	0.329

log_IL3	0.000	0.997	-0.280	0.279	-0.144	0.354	-0.453	0.165
log_IL12p40	0.009	0.907	-0.150	0.168	-0.033	0.705	-0.210	0.143
log_IL16	-0.121	0.569	-0.543	0.301	-0.086	0.698	-0.526	0.355
log_CTACK	-0.066	0.398	-0.221	0.089	-0.093	0.237	-0.248	0.063
log_GROa	-0.027	0.899	-0.459	0.404	-0.123	0.573	-0.557	0.312
log_HGF	-0.086	0.593	-0.409	0.236	0.014	0.937	-0.334	0.361
log_IFNa2	-0.101	0.620	-0.507	0.305	-0.286	0.194	-0.723	0.151
log_LIF	-0.107	0.452	-0.388	0.175	-0.200	0.174	-0.491	0.091
log_MCP3	-0.054	0.701	-0.335	0.227	-0.112	0.467	-0.419	0.195
log_MCSF	-0.159	0.217	-0.414	0.096	-0.121	0.388	-0.399	0.158
log_MIF	-0.143	0.331	-0.434	0.149	-0.085	0.568	-0.384	0.213
log_MIG	-0.012	0.917	-0.237	0.213	-0.037	0.746	-0.262	0.189
log_NGF	-0.036	0.827	-0.363	0.291	-0.266	0.150	-0.630	0.099
log_SCF	-0.199	0.277	-0.563	0.165	-0.242	0.204	-0.620	0.136
log_SCGFb	-0.244	0.058	-0.496	0.008	-0.211	0.103	-0.466	0.044
log_SDF1a	0.430	**0.029**	0.047	0.814	0.248	0.365	-0.297	0.793
log_TNFb	-0.064	0.518	-0.260	0.133	-0.078	0.444	-0.281	0.125
log_TRAIL	-0.078	0.432	-0.275	0.119	-0.058	0.571	-0.263	0.147

- Dependent variable: CD4:CD8 ratio; both models included study arm; multivariable analysis adjusted for contraception, age, study site, HSV-2 at baseline and study arm.
- p-values that passed the FDR correction are indicated with a *

Sup. Table 5. Bivariate and multivariable survival analysis of the effect of pre-infection plasma cytokines on CD4 decline <500µl

Variables	HR (95% CI)	p-value	aHR *(95% CI)	p-value	aHR (-VL)*(95% CI)	p-value
Log IL-β	0.616 (0.175-2.167	0.450	0.452 (0.128-1.587)	0.215	0.597 (0.161-2.219)	0.442
Log IL-1rα	1.001 (0.239-4.201)	0.999	0.443 (0.096-2.051)	0.298	1.069 (0.243-4.697)	0.929
Log IL-2	0.936 (0.418-2.094)	0.871	0.318 (0.109-0.926)	**0.036**	0.905 (0.368-2.225)	0.828
Log IL-4	0.535 (0.168-1.698)	0.288	0.562 (0.178-1.779)	0.327	0.521 (0.159-1.709)	0.282
Log IL-5	0.790 (0.389-1.605)	0.514	0.692 (0.336-1.429)	0.320	0.847 (0.418-1.716)	0.644
Log IL-6	0.858 (0.301-2.444)	0.774	0.441 (0.151-1.286)	0.134	0.829 (0.286-2.405)	0.730
Log IL7	0.584 (0.247-1.378)	0.219	0.544 (0.222-1.334)	0.184	0.574 (0.236-1.399)	0.222
Log IL-8	0.823 (0.183-3.698)	0.800	0.422 (0.068-2.628)	0.355	0.733 (0.145-3.705)	0.707
Log IL-9	0.589 (0.229-1.515)	0.272	0.527 (0.196-1.413)	0.203	0.569 (0.221-1.465)	0.242
log IL-10	1.109 (0.743-1.656)	0.613	0.592 (0.349-1.005)	0.052	1.046 (0.657-1.666)	0.850
Log IL-12p70	0.995 (0.580-1.707)	0.985	0.522 (0.251-1.089)	0.083	0.831 (0.424-1.626)	0.589
Log IL-13	0.803 (0.338-1.908)	0.620	0.534 (0.210-1.355)	0.187	0.723 (0.276-1.894)	0.509
Log IL-15	0.762 (0.373-1.556)	0.456	0.682 (0.296-1.568)	0.367	0.839 (0.385-1.831)	0.660
Log IL-17	0.880 (0.451-1.719)	0.708	0.675 (0.319-1.431)	0.306	0.852 (0.424-1.713)	0.654
Log Eotaxin	0.564 (0.219-1.451)	0.235	0.495 (0.155-1.569)	0.232	0.604 (0.211-1.728)	0.347
Log FGF basic	0.750 (0.440-1.279)	0.290	1.236 (0.590-2.589)	0.574	0.640 (0.327-1.252)	0.193
Log GCSF	0.357 (0.107-1.196)	0.095	0.404 (0.116-1.408)	0.155	0.377 (0.108-1.313)	0.126
Log GMCSF	0.959 (0.483-1.903)	0.904	1.106 (0.529-2.313)	0.788	0.881 (0.420-1.850)	0.738
Log IFNy	1.027 (0.521-2.022)	0.939	0.902 (0.476-1.708)	0.750	1.017 (0.500-2.068)	0.962
Log IP-10	0.689 (0.301-1.580)	0.379	0.746 (0.247-2.253)	0.604	0.645 (0.213-1.947)	0.436
Log MCP-1	1.073 (0.519-2.220)	0.849	0.732 (0.313-1.716)	0.473	0.990 (0.441-2.221)	0.980
Log MIP-1α	0.312 (0.071-1.379)	0.125	0.255 (0.034-1.900)	0.183	0.213 (0.031-1.457)	0.115
Log PDGF-ββ	0.688 (0.398-1.190)	0.181	0.759 (0.426-1.354)	0.350	0.684 (0.385-1.215)	0.195
log MIP-1β	2.369 (0.630-8.911)	0.202	3.882 (0.675-22.339)	0.129	4.079 (0.957-17.391)	0.057
Log RANTES	0.944 (0.356-2.501)	0.908	1.090 (0.394-3.012)	0.868	0.939 (0.332-2.655)	0.906
Log TNFα	0.709 (0.277-1.817)	0.474	0.657 (0.262-1.648)	0.370	0.744 (0.276-2.008)	0.559
Log VEGF	O.726 (0.229-2.304)	0.587	0.375 (0.087-1.624)	0.190	0.557 (0.139-2.231)	0.409
Log IL-1α	11.513 (0.532-249.016)	0.119	9.768 (0.424-224.822)	0.154	8.564 (0.378-194.094)	0.177
Log IL-2ra	0.838 (0.155-4.547)	0.838	0.560 (0.082-3.814)	0.553	1.273 (0.188-8.635)	0.805
log IL-3	0.866 (0.248-3.022)	0.822	0.268 (0.069-1.039)	0.057	1.083 (0.269-4.359)	0.910
Log IL-12p40	1.234 (0.688-2.215)	0.480	0.563 (0.282-1.124)	0.104	1.345 (0.695-2.600)	0.379
log IL-16	4.546 (0.919-22.484)	0.063	0.988 (0.154-6.326	0.990	4.595 (0.907-23.270)	0.065
Log CTACK	1.246 (0.686-2.264)	0.470	0.875 (0.435-1.760)	0.708	1.518 (0.760-3.032)	0.237
log GROα	0.387 (0.081-1.846)	0.234	0.131 (0.018-0.965)	**0.046**	0.416 (0.084-2.051)	0.281
Log HGF	2.036 (0.557-7.441)	0.282	0.885 (0.199-3.938)	0.872	1.919 (0.489-7.522)	0.350
log IFNα2	1.208 (0.232-6.287)	0.822	0.118 (0.016-0.847)	**0.034**	1.663 (0.277-9.970)	0.578
Log LIF	1.408 (0.457-4.340)	0.551	0.552 (0.159-1.911)	0.348	1.935 (0.613-6.111)	0.261
Log MCP-3	1.434 (0.540-3.804)	0.469	0.303 (0.078-1.176)	0.084	1.799 (0.605-5.354)	0.291
Log MCSF	1.253 (0.434-3.616)	0.676	0.501 (0.137-1.829)	0.296	1.042 (0.317-3.427)	0.946
Log MIF	0.696 (0.255-1.901)	0.480	0.484 (0.144-1.619)	0.239	0.672 (0.242-1.867)	0.446
Log MIG	1.737 (0.801-3.765)	0.162	1.060 (0.450-2.499)	0.894	1.820 (0.844-3.924)	0.126
Log NGF	0.792 (0.214-2.929)	0.727	0.430 (0.095-1.956)	0.275	1.052 (0.235-4.701)	0.947
Log SCF	3.147 (0.714-13.880)	0.130	0.363 (0.071-1.862)	0.224	2.928 (0.672-12.747)	0.152
Log SCGFβ	3.712 (1.279-10.771)	**0.016**	2.738 (0.729-10.290)	0.136	4.537 (1.475-13.954)	**0.008**
Log SDF-1α	0.666 (0.159-2.792)	0.578	0.087 (0.009-0.853)	**0.036**	1.082 (0.145-8.076)	0.939
log TNFβ	2.236 (1.125-4.445)	**0.022**	1.745 (0.813-3.746)	0.153	2.318 (1.133-4.741)	**0.021**
log TRAIL	0.728 (0.303-1.751)	0.479	0.446 (0.196-1.015)	0.054	0.641 (0.247-1.660)	0.360

- Study arm was included in all models; multivariable analysis adjusted for contraception, age, study site, HSV-2 at baseline study arm, +/- VL
- p-values that passed the FDR correction are indicated with a *
